# Increasing Dietary Carbohydrate as Part of a Healthy Whole Food Diet Intervention Dampens Eight Week Changes in Salivary Cortisol and Cortisol Responsiveness

**DOI:** 10.3390/nu11112563

**Published:** 2019-10-24

**Authors:** Hoda Soltani, Nancy L. Keim, Kevin D. Laugero

**Affiliations:** 1 Department of Nutrition, University of California, Davis, CA 95616 1, USA; hsoltani@ucdavis.edu (H.S.); nancy.keim@ars.usda.gov (N.L.K.); 2 Obesity and Metabolism Research Unit, USDA/ARS/Western Human Nutrition Research Center 2, Davis, CA, 95616, USA

**Keywords:** diet intervention, dietary guidelines, dietary carbohydrate, salivary cortisol, Trier Social Stress Test

## Abstract

It is largely unknown whether and how whole food diets influence psychological stress and stress system responsiveness. To better understand the effects of whole diets on stress system responsiveness, we examined randomized control trial effects of a whole food diet based on the Dietary Guidelines for Americans (DGA) on cortisol responsiveness. A randomized, double-blind, controlled 8-week intervention was conducted in overweight and obese women to examine differentiated effects between two diet intervention groups: one based on the 2010 DGA and the other one based on a typical American diet (TAD). During a test week that occurred at baseline and again after 8 weeks of the intervention, we assessed salivary cortisol collected at 14 selected times across the day, including upon awakening, at bedtime, and during a test visit, and administered a standardized social stress task (Trier Social Stress Test, TSST). There were no statistical differences between the diet groups in salivary cortisol at baseline or after 8 weeks. However, when considering differences in dietary carbohydrate, but not fat or protein, from the pre-intervention (habitual) to the intervention period, there was a significant (*P* = 0.0001) interaction between diet group, intervention week, saliva sample, and level of intervention-based change in carbohydrate consumption. This interaction was reflected primarily by an 8-week reduction in salivary cortisol during a period just prior to (log Δ −0.35 ± 0.12 nmol/L) and 30 (log Δ −0.49 ± 0.12 nmol/L), 60 (log Δ −0.50 ± 0.13 nmol/L), 90 (log Δ −0.51 ± 0.13 nmol/L), and 120 (log Δ −0.4476 ± 0.1231 nmol/L) min after the TSST in the DGA group having the highest increase (90th percentile) in carbohydrate consumption. In support of this finding, we also found significant (*P* < 0.05) and inverse linear associations between dietary carbohydrate and log salivary cortisol, with the strongest negative association (β: −0.004 ± 0.0015, *P* = 0.009) occurring at 30 min post-TSST, but only in the DGA group and at week 9 of the intervention. Together, increasing dietary carbohydrate as part of a DGA-based diet may reduce circulating cortisol and dampen psychological stress-related cortisol responsiveness.

## 1. Introduction

While a healthy diet is often recommended as a strategy for managing stress and stress-related diseases, there are no evidence-based, specific dietary guidelines that target such concerns. There is a growing awareness about the advantages of nutritional medicine in psychiatry [1], prompting an increased focus on the interrelationships between stress, mood, and nutrition. Several studies have shown the influence of nutrients and specific foods on an individual’s physiological, neural, and psychological stress response [2,3,4,5,6,7,8,9,10,11,12,13,14,15,16]. For example, high-fat feeding increases circulating corticosterone in rodent models [17,18], and high-fat meals were shown to exacerbate detrimental autonomic nervous systems and cardiovascular responses to stress [8]. In contrast, polyunsaturated fat intake can diminish stress-induced cardiovascular responses [3], omega-3 fatty acid consumption normalized abnormally low cortisol responses to an acute stress test [6], and milk-based phospholipids improved memory in men reporting high levels of chronic stress [14]. In addition, adding egg powder to the diet normalized the endocrine and the negative emotional response to stress, which thereby normalized neuroendocrine stress responses and reduced adaptation to acute stress [15]. 

It was also shown that consumption of fermented foods is associated with reduced social anxiety [19]. Finally, dietary sugar or carbohydrate may dampen stress-induced elevation in glucocorticoid hormones, such as corticosterone (rodents) and cortisol (human) [20,21,22,23,24], a phenomenon thought to physiologically explain and strengthen comfort eating or the stress–eating relationship [25,26]. However, little is known about how whole food diets, such as those based on the Dietary Guidelines for Americans (DGA), may impact daily cortisol and cortisol responsiveness. 

Despite the potential for whole food diets, such as those based on DGA standards, to mitigate the magnitude of stress reactions, DGA adherence is typically poor [27,28]. Furthermore, diet interventions intended to diminish cardiometabolic issues are often limited by individuals’ dietary and weight relapse in the long term [29]. This is unfortunate given the fact that those who can sustainably adopt a diet based on DGA recommendations have a diminished risk for developing obesity-related diseases, such as type 2 diabetes [30,31,32]. Regression to previous eating behaviors is linked to neurological factors associated with psychological, emotional, and cognitive functions [33,34,35,36]. In fact, adopting new dietary patterns can trigger psychological stress and its associated increases in cortisol [37,38] which, in turn, can limit progress in making these dietary changes sustainable [39,40] and thereby limit the overall effectiveness of dietary recommendations. 

Psychological stress can adversely influence the quality of food choices, weight loss, and metabolic health [41]. Feelings of stress can stimulate desire to consume highly palatable, calorically dense food and simultaneously disturb self-regulation and goal-directed decision-making by diminishing the cognitive processes that underlie them [42]. Reward-based eating and reactionary eating can be heightened by disruptions of homeostatic regulation of food intake induced by stress [43,44]. The ability to self-regulate behaviors and consciously make choices that favor long-term outcomes over short-term rewards is important for supporting long-term health and dietary behavior changes, particularly in the context of stress [45]. Given reports suggesting the increasing prevalence of stress [46,47] and availability of highly palatable but nutritionally poor foods [48,49], it is important to evolve the current understanding of stress networks controlling motivation. Such an understanding may assist in developing effective nutrition and disease prevention messaging and hopefully translate to assisting the public in making meaningful and durable improvements to their diet, irrespective of life’s daily hassles. 

To better understand the effects of whole diets on daily cortisol and cortisol responsiveness, we examined, in a randomized control trial, effects of a DGA-based diet on salivary cortisol throughout the day and in response to a standardized stress test, the Trier Social Stress Test. Exploratory analyses were also performed to assess if the magnitude of departure from the participants’ usual pre-intervention dietary carbohydrate, fat, and protein mediated the effects of the intervention diet on salivary cortisol. A primary report on the effect of the intervention regarding clinical risk factors for type 2 diabetes and cardiovascular disease was recently published [50].

## 2. Materials and Methods

### 2.1. Study Design

This report was derived from a secondary analysis based on women who participated in a study reported by Krishnan et al. [50], in which the whole food diet consumption effects on cardiometabolic risk factors were measured. In this study, a randomized, double-blind, controlled 8-week fed intervention was conducted in overweight and obese women randomly assigned to one of two diet groups: a diet based on the 2010 DGA or a diet based on a typical American diet (TAD). Both DGA- and TAD-based diets were isocaloric and developed to maintain body weight over the 8-week intervention, while providing commonly available foods and beverages. The diets varied primarily in carbohydrates and sources of fat, with the DGA diet (56% carbohydrates, 18% protein, and 26% fat) containing more whole grains, low-fat dairy, polyunsaturated fat, vegetables, and fruit, while the TAD-based diet (52% carbohydrates, 15% protein, and 34% fat) included more added sugars, refined grains, and solid and total fat. Krishnan et al. [50] published specific nutrient breakdown, sample menus, and key products used to prepare meals for each diet. 

Study participants were informed that the research was investigating a broad set of factors that may contribute to understanding dietary effects on the body. Following orientation to the study, subjects were examined over a 1-week baseline (pre-intervention) period, during which they continued to consume their usual diets. This pre-intervention period was followed by the 8-week, meal-controlled diet intervention. During the pre-intervention period, physical activity, usual diet, energy requirements, body composition, cardiometabolic risk markers, and markers of psychological and physiological stress were estimated. These measurements were repeated at the end of the 8-week intervention. A detailed description of the study timeline and measurements performed in this study can be found in the publicatio-n by Krishnan et al. [50]. The trial was conducted at the Western Human Nutrition Research Center (WHNRC) in Davis, CA. The study was approved by Institutional Review Board at the University of California, Davis, and informed consent was given by subjects prior to participating in the study. This trial was approved by Davis Institutional Review Board in the University of California and is registered (NCT02298725) at clinicaltrials.gov. All participants provided written informed consent for participating in the study. 

### 2.2. Subjects

Women, who were overweight or obese, aged 20–64, had a body mass index (BMI) of 25–39.9 kg/m^2^, displayed either insulin resistance and/or dyslipidemia, and did not meet the minimal physical activity guidelines of 150 min/week, were recruited for the study. To determine eligibility during the screening process, study staff completed phone interviews where they verbally administered a telephone screening questionnaire. Potential participants were excluded if they were identified with any of the following characteristics: presence of any metabolic diseases, gastrointestinal disorders, cancer, or other serious chronic disease; pregnancy or lactating; current use of tobacco; prescribed or over-the-counter weight-loss medications in 6 months before enrollment into the study; moderate or strenuous physical activity of >30 min/day on no less than 5 days per week; weight change of >5% of body weight within 6 months of entry into the study; resting blood pressure of >140/90 mm Hg, hemoglobin of <11.5 g/dL, total cholesterol of >300 mg/dL, Low Density Lipoprotein (LDL) cholesterol of >189 mg/dL, triglycerides of >400 mg/dL, and clinically abnormal thyroid or liver function; “graveyard” work shifts or forced to stay awake all night; dietary restrictions that would interfere with consuming the intervention foods; or use of corticosteroids and medications for elevated lipids or glucose. In addition, individuals considered to be vulnerable, including adults unable to consent, infants, children, and prisoners, were not eligible for the study. 

### 2.3. Measurements

#### 2.3.1. Dietary Measurements

To approximate typical diet quality, subjects completed 24 h dietary recalls using an automated and self-administered system [51] during the pre-intervention period. Three unannounced 24 h dietary recalls were administered wherein participants input their intake for the previous 24 h. Once entered, dietary data were quantified into specific nutrient and food categories, including the major macronutrients carbohydrate, fat, and protein, each of which was shown to influence stress-associated glucocorticoid concentrations [17,52,53]. Success in dieting or adopting new dietary habits may depend on an individual’s eating behavior characteristics, making them more or less vulnerable to successful behavioral change. For example, certain characteristics such as restrained eating and disinhibition have been associated with increases in circulating concentrations of cortisol [54,55,56]. To help account for these eating behavior characteristics, we used the Three-Factor Eating Questionnaire (TFEQ) to assess hunger, restrained eating, and disinhibition. The TFEQ asks a series of questions to measure individuals’ relationship to eating with respect to the dimensions of restraint (21 items), disinhibition (16 items), and hunger (14 items). The multiple-choice questionnaire asks about typical eating behaviors, and responses are scored 0 or 1 and summed. High scores indicate higher levels of disordered eating as it relates to restraint, disinhibition, and feelings of hunger, based on each respective section of the questionnaire [57]. 

#### 2.3.2. Salivary Cortisol Measurements 

Saliva was collected at home upon waking (saliva sample 1), 30 min post-waking (saliva sample 2), and at bedtime (saliva sample 14). Saliva was also collected 11 times during a test visit on study weeks 1 and 9. These samples were collected upon arrival (saliva sample 3) at the research center and at 30 min intervals after a morning oral glucose tolerance test (saliva samples 4–7), in the afternoon after a standard lunch (saliva samples 8 and 9), and then at 30 min intervals after the induction of the Trier Social Stress Test (TSST, saliva samples 10–13). Sample number 9 was taken immediately before initiating the stress test and served as the pre-TSST sample. The samples were collected using Salimetrics Oral Swabs. Participants put the swab in their mouth for 1–2 min in order to collect saliva and then deposited the swab into provided sample tubes. The sample tubes collected at home, after waking and before going to bed, were stored in the participant’s refrigerator prior to returning the samples to the WHNRC for analysis, using an ELISA method (expanded-range high-sensitivity salivary cortisol kit, Salimetrics, State College, PA), which can detect cortisol concentrations ranging from 0.193 to 82.77 nmol/L (0.007–3.0 μg/dL). The intra- and inter-assay coefficients of variability are 3.5% and 5.1%, respectively. 

To assess acute stress-related cortisol responsiveness, the TSST was administered for about 2 h after eating a standard lunch at each test visit on weeks 1 and 9. The TSST consists of two challenging tasks known to evoke moderate psychological stress and increases in plasma and salivary cortisol [58]: a speech task and a mental arithmetic task. Both of these tasks were performed in front of two judges (they were unfamiliar to the subjects) and a video camera (subjects were told that videos were retained and used only by research staff to evaluate the participant’s behavior during the task). Following a rest period, participants were brought into a room and introduced to the judges who told them about the tasks they would be performing over the next 20 min. Each subject was then be given 5 min to prepare her 5 min speech, after which time the judges would return and the speech would begin. Directly following the speech was a 5 min question-and-answer session, in which the subject was asked about her past work experience and experiences with managers and co-workers. After this session, subjects were given 5 min to count backwards in odd steps from a prime number (e.g., starting at 1022 and counting backwards by 13) as quickly and accurately as possible. If a mistake was made, the subject was asked to start from the beginning.

#### 2.3.3. Physical Measurements

Measurements of height, weight, and waist-to-hip ratio were collected to provide information about participants’ anthropometric status. Participants were weighed wearing lightweight surgical scrubs using a calibrated electronic scale (Tanita BWB-627A Class III electronic scale; Toledo Scale), which were measured to the nearest 0.1 kg. A wall-mounted stadiometer (model S100; Ayrton Corporation, Prior Lake, MN, USA) was used to measure the height to the nearest 0.1 cm. These values were used to calculate BMI with the unit of kg/m^2^. Waist and hip circumferences were measured in duplicate for accuracy with an anthropometric tape around the circumference of the area between the iliac crest and the rib cage for waist circumference, and at the maximum protuberance of the buttocks for hip circumference. A standard blood pressure cuff (GE DINAMAP vitals monitor; GE Healthcare, Chicago, IL, USA) placed on one arm was used to measure blood pressure.

### 2.4. Statistical Analysis 

The SAS System software for Windows (release 9.4; Cary, NC, USA) was used to perform statistical analyses. Salivary cortisol concentrations were logarithm-transformed. To examine effects of the intervention on log salivary cortisol, we first applied a mixed model procedure (SAS MIXED procedure), which included fixed effects of diet group (DGA vs. TAD, intervention week (baseline (week 1) and week 9), saliva sample (1–14), all 2- and 3-factor interactions of the fixed effects, along with random effects of subject, subject by week, and subject by saliva sample to account for repeated measurements across the intervention week and the 14 saliva samples. Significant interactions between group and intervention week, and group, intervention week, and saliva sample were considered indicative of intervention effect. Fourteen subjects from each diet group completed both stress tasks (week 1 and week 9). However, saliva was still collected during all 14 time points on week 1 and week 9 from those subjects not participating in the stress task. Therefore, consistent with the intention-to-treat approach, we used all of the study participants in the analysis, except where noted as stated below. Baseline age, BMI, and education level were included as independent variables in the model. Because we also found baseline hunger (TFEQhunger), but not restrained eating or disinhibition from the TFEQ, to positively associate with salivary cortisol, we then also included the hunger score from the TFEQ as an independent variable in the final statistical model. Because one participant in the TAD group did not complete the baseline TFEQ, her data were dropped from the statistical analysis. Therefore, in the final model that included hunger, there were 22 subjects in the DGA group and 21 in the TAD group. Since the difference between the pre-intervention (“habitual”) diet and the intervention diet (DGA; TAD) differed in magnitude for each subject, we hypothesized that these differences in dietary macronutrient shifts may possibly moderate the effects of the intervention on 8-week change in salivary cortisol and cortisol responsiveness. To explore whether intervention-based shifts in carbohydrate, protein, and fat consumption might moderate the effects of the diet intervention on salivary cortisol, we applied the above statistical model, but also included, one at a time, the intervention-based change in each of these macronutrients and their interactions with diet group, intervention week, saliva sample in the model. We observed a significant 4-factor interaction between diet group, intervention week, saliva sample, and change in carbohydrate intake, suggesting that effects of diet depended not only on week and saliva sample, but also on the level of change in carbohydrate intake. To better understand the nature of this interaction, we estimated the expected value of log cortisol for each combination of diet group, intervention week, and saliva sample at selected levels of change in carbohydrate (mean and 90th and 10th percentiles across all subjects), using the least-square means (LSMEANS) option in SAS. To statistically assess the nature of this interaction, from the same model, we checked for statisically significant (t-test_slope different from 0_, *P* < 0.05) associations between log salivary cortisol (nmol/L) and change in carbohydrate consumption. Using this same model, we also followed this examination up with specific contrasts to also test for significantly (t-test_,_ *P* < 0.05) different regression coefficients (slopes) between diet groups at each combination of intervention week and saliva sample. 

## 3. Results

**Figure 1:**
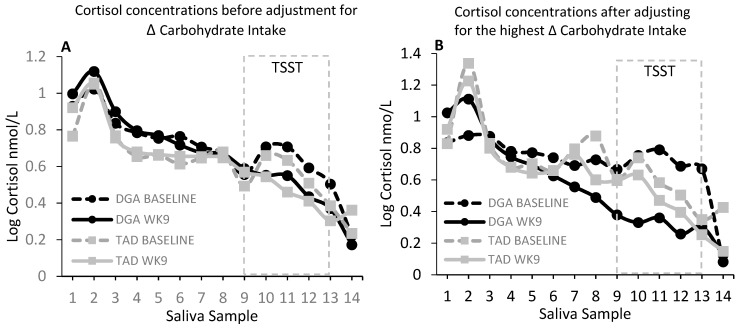

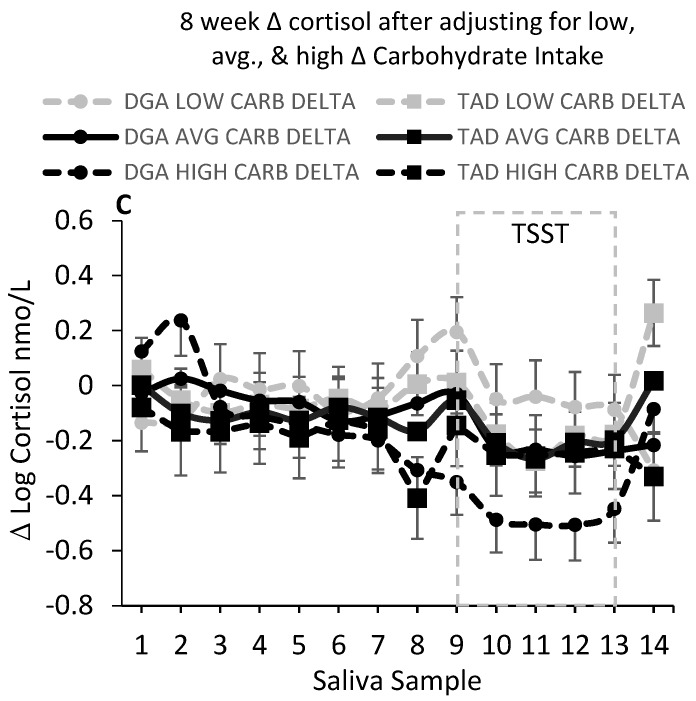
(**A**) shows salivary cortisol (nmol/L) at baseline (pre-intervention test week 1) and week 9 for each diet group at each time point from waking (sample 1) to bedtime (sample 14). There were no statistical differences in salivary cortisol between the diet groups (DGA, *N* = 22; TAD, *N* = 21). However, when accounting for inter-individual differences between the pre-intervention (“habitual”) diet and the intervention diet, there was a significant (*P* = 0.0001) interaction between diet group, week, saliva sample, and magnitude of change in carbohydrate intake from habitual to the intervention level of intake. The significant (*P* = 0.0001) interaction between diet group, week, saliva sample, and level of change in carbohydrate intake was reflected primarily by a reduction in salivary cortisol just prior to the stress test (Trier Social Stress Test; TSST) and up to 120 min post-stress in the DGA (Dietary Guidelines for Americans) group that also had the highest increase (90th percentile) in carbohydrate consumption from the pre-intervention to the intervention period. (**B**) Salivary cortisol (nmol/L) at baseline and week 9 for each diet group at the highest increase (90th percentile) in carbohydrate consumption from the pre-intervention (habitual) to the intervention period. (**C**) The 8-week change (Δ) in salivary cortisol concentration (nmol/L). There was a significant (*P* = 0.0001) interaction between diet group, saliva sample, and level of change in total carbohydrate intake for the 8-week change (Δ) in salivary cortisol concentration. A low increase in CARB (total carbohydrate; +166 g) was taken as the 10th percentile increase in carbohydrate consumption from the pre-intervention to the intervention period; an AVG (average; middle 90th percentile) increase in carbohydrate (+224 g) was taken as the mean level increase in carbohydrate consumption from the pre-intervention to the intervention period; and a high increase in CARB was taken as the 90th percentile increase in carbohydrate (+305 g) from the pre-intervention to the intervention period. Baseline age, education, body mass index (BMI), and hunger score from the Three-Factor Eating Questionnaire were included as independent variables in all of the statistical models. For visual clarity, data shown in Figure 1A,B are adjusted means (least-square means; LSMEANS) only. For Figure 1C, LSMEANS ± s.e. are presented.

In this randomized control diet intervention, 8-week change in salivary cortisol, examined over 14 daily time points throughout the day, did not appear to be affected by the diet intervention. We did not find a significant (*P* > 0.05) main effect of diet or diet–week, diet–saliva sample number, or diet–week–saliva sample interactions (Figure 1A). However, the intervention-based change in carbohydrate consumption appeared to moderate the effects of the diet intervention on salivary cortisol concentrations, particularly the cortisol concentrations just prior to and up to 120 min after the induction of the TSST (Figure 1B,C). Significant results were amplified when adding the hunger score from the TFEQ to the model. We found a significant (*P* = 0.0001) 4-factor interaction between diet, intervention week, saliva sample number, and intervention-based change in carbohydrate consumption. To examine the nature of this interaction, we evaluated the salivary cortisol at low (10th percentile; +166 g), average (+224 g), and high (90th percentile; +305 g) levels of change in carbohydrate consumption. As shown in Figure 1B,C, it is evident that this interaction reflected a DGA-associated reduction in salivary cortisol at week 9 when the intervention led to higher (90th percentile) increases in the consumption of this macronutrient (Figure 1B). Figure 1C displays the 8-week change (Δ) in cortisol at each time point and for each of the low (10th percentile), mean, and high (90th percentile) levels of intervention-based changes in carbohydrate consumption. In support of these findings, we found significant inverse associations between log cortisol and change in carbohydrate in the DGA group, only at week 9 of the intervention, and at specific times during the day (Table 1). Similar to the above observations, these associations were present at times just prior to and up to 90 min after the stress test. The strongest and most significant association between log cortisol and change in carbohydrate occurred at the 30-min stress response time point (β: −0.004 ± 0.0015, *P* = 0.009). We also found that this association at week 9 and the 30-min stress response time point significantly (*P* = 0.0236) differed between the DGA and TAD groups.

## 4. Discussion

### 4.1. Differences in Dietary Carbohydrate Moderate Effects of the Diet Intervention on Salivary Cortisol

Stress, stress-induced increases in circulating cortisol, and elevations in basal circulating cortisol are linked to disorders of mood and cognitive decline [58,59,60,61,62], and these effects may, in part, result from the propensity of stress and elevated cortisol concentrations to promote poor nutrition habits and reduce adherence to health-promoting diets, such as those based on the DGA. Therefore, such dietary effects of stress and cortisol can significantly limit the effectiveness of dietary recommendations in treating or preventing diseases such as type 2 diabetes. Furthermore, it is widely accepted that a healthy diet can play an important role in the management of cognitive, neuroendocrine, and autonomic responses to stress and consequently play an important role in the prevention of depression and other stress related diseases. Given this, it is surprising that specific, evidence-based, dietary recommendations for supporting stress management do not exist. This study is the first of its kind to utilize a randomized control trial model to compare effects of consuming DGA- or TAD-based diets on cortisol and stress-related cortisol responsiveness. In particular, very little is known about how whole food diets, such as those based on the DGA, influence circulating cortisol.

There were no statistical differences between the diet groups in salivary cortisol at baseline or after 8 weeks. However, since the difference between the pre-intervention (“habitual”) diet and the intervention diet (DGA; TAD) differed in magnitude for each subject, we hypothesized that the degree of change in dietary macronutrient differences may possibly moderate the effects of the intervention on 8 week change in salivary cortisol and cortisol responsiveness. When considering these differences in dietary carbohydrate from the pre-intervention (habitual) to the intervention period, our current findings suggest that increasing dietary carbohydrate, as part of a healthy whole food diet, dampens 8-week changes in salivary cortisol, particularly during the period following the stress test. We did not observe this effect with dietary fat or protein.

Several studies have shown dietary sugar or carbohydrate to reduce stress-induced elevation in glucocorticoid hormones, such as corticosterone (rodents) and cortisol (human) [20,21,22,23,24], a phenomenon thought to physiologically explain and strengthen comfort eating or the stress-eating relationship [25,26]. A diet high in carbohydrate was shown to reduce cortisol and negative mood after stress [63], and carbohydrate loading was shown to increase performance and inhibit the typical cortisol increase in response to prolonged exercise [64]. Given the physiological link between the glucocorticoids and glucose regulation and preference for dietary carbohydrate, it is not surprising to observe an association between circulating cortisol and changes in carbohydrate consumption. However, our study uniquely suggests that these apparent effects of carbohydrate consumption may interact with the quality of the total diet being consumed. It is interesting that including the TFEQ hunger score in the interaction resulted in an amplified moderating effect of carbohydrate differentiation in stress response between the two diet groups. This may suggest that carbohydrate intake may have an attenuating effect on hunger which can, in turn, increase susceptibility to stress. We did not observe these dampening effects of increasing carbohydrate in the context of the TAD. The TAD used in this study was higher in saturated fat [50]. Previous studies have shown inductive effects of a high-fat diet on stress responsiveness including cortisol and corticosterone [8,17,18]. It is possible, therefore, that this inductive effect of increasing certain types of dietary fat may have countered the dampening effects of increasing carbohydrate intake on cortisol in the TAD group. 

Alternatively, it was recently shown in a cross-sectional study of adolescents that higher adherence to a Mediterranean diet resulted in reduced inflammation and a disassociation between inflammatory cytokines and salivary cortisol [65]. Since high-fat diets can induce mild inflammation [66], it is possible the DGA diet provided a similar protective effect against inflammation and inflammatory-induced cortisol [67], compared to the higher-fat TAD. However, while the TAD was higher in saturated fat, we did not observe an association between salivary cortisol and the intervention-based change in dietary fat. A higher sodium consumption in the TAD may have also masked the cortisol lowering effects of increasing carbohydrate. Prior research suggests that increasing dietary sodium can elevate circulating cortisol. We previously showed that increasing sodium consumption is positively associated with markers of chronic stress, including higher 12 h, overnight, urinary cortisol concentrations [68]. There also exists other evidence that increases in sodium intake or salt load can raise urinary cortisol [69,70,71], while restricting sodium intake appeared to reduce urinary cortisol [72]. On the other hand, there may have been a more permissive effect of the DGA diet itself, possibly through the effects of other nutrients on neurological systems that regulate cortisol production. For example, dietary vitamin D, which was higher in the DGA group [50], was shown to facilitate ongoing serotonergic activity in the brain [73], possibly by suppressing the serotonin reuptake transporter and inhibiting serotonin reuptake. The stress and cortisol reducing effects of carbohydrate consumption have also been thought to be mediated, in part, by enhanced serotonergic activity in the brain [74], which also may enhance negative feedback in the hypothalamic–pituitary–adrenal axis and reduce circulating cortisol [62,74,75]. These explanations are only speculative, but our results warrant mechanistic studies to determine how and why changes in dietary carbohydrate link to alterations in cortisol in the context of the DGA-based, but not TAD-based, whole food diets.

Given the known detrimental health risks of chronic stress load, in part through heightened exposure to cortisol, these findings may have important clinical implications. Increases in cortisol and stress-associated cortisol responsiveness have been linked to elevated risk for central obesity, metabolic syndrome, cardiovascular disease, type 2 diabetes, depression, and other disorders of the brain [60,76]. Therefore, as it relates to managing unhealthy stress reactions and its effects on mental and metabolic health, it may be appropriate to consider carbohydrate intake, and perhaps even increasing intake in the context of a healthy whole food diet. Of course, in some cases, such as in diabetics, increasing carbohydrate consumption is not recommended. Our findings also suggest that, in the context of dieting or adopting a healthier diet, carbohydrate consumption may help to minimize dietary relapse due to the stress of restricting favorite, highly palatable comfort foods [24,25,77]. Chronic stress and over-exposure to the glucocorticoid hormone, cortisol, can increase preference for and drive to eat highly palatable, nutrient scarce foods [37], thereby reducing in some persons capacity for sustaining significant and durable improvements in diet quality. Chronic dieting and repeated attempts to avoid preferred foods, even with unsuccessful weight loss, has been shown to be associated with elevations in stress markers, and salivary and urinary cortisol, independent of BMI [54,55,78]. Our findings suggest that carbohydrate consumption as part of a healthy whole food diet may help minimize over-exposure to cortisol and its associated downstream effects, including increasing hunger for comfort foods. This has implications for individuals consuming highly carbohydrate-restrictive diets or individuals, for whom stress presents a significant barrier to consuming a whole food diet overall. Since the variable CARB represents degree of change from habitual diet, the results suggest that the degree of change to carbohydrate intake may affect circulating cortisol levels. Therefore, diets that include a drastic reduction in carbohydrates may precipitate stress-inductive effects. These results support the notion that carbohydrate-restrictive diets may have stress-inducing effects that may work counter to their ability to be sustainable. In these cases, if possible, it may be worthwhile incrementally increasing carbohydrate intake, if possible, to alleviate any stress-inductive effects. 

### 4.2. Limitations

To our knowledge, this is the first controlled-feeding trial comparing the effects of a whole food diet with a more representative American diet on salivary cortisol and stress-related cortisol responsiveness. However, we acknowledge limitations associated with this trial, as previously reported [50]. The 8-week duration of this study is, to some degree, short and may not represent effects, or lack thereof, that may be observed over a longer period of time. In addition, we acknowledge that our study included a relatively small sample size. It should also be re-emphasized that, while our results suggest that the carbohydrate intake and diet group interacted to affect stress-related cortisol concentrations or responsiveness, we acknowledge that this diet group specific association between carbohydrate and cortisol during the week-9 stress test period cannot definitively be assumed to be linked to the stress response, since a subset of the participants did not participate in the stress test on both visits. Finally, as this study was conducted in women, it is important to note that our findings may not be generalized for men or individuals with normal glucose or lipid values. 

## 5. Conclusions

This study provides further evidence for the health benefits of a DGA-based whole food diet. Furthermore, we add to a growing body of evidence supporting the potential effects of suppressive effects of dietary carbohydrate on circulating cortisol and stress-associated cortisol reactivity. 

Such new information may help to inform an understanding of nutrition where key food groups facilitate improved adherence to often fleeting healthy changes in diet. Overall, these results support novel target areas for improving nutrition and disease prevention messaging. 

## Figures and Tables

**Table 1:** * Slope estimates for association between log salivary cortisol concentration and Δ carbohydrate.

Sample Time (Sample Number)	Diet	Intervention Week	Slope	S.E.	*P*-Value
Pre-TSST (9)	DGA	Baseline	0.001463	0.001487	0.3266
	DGA	9	−0.00312	0.001487	**0.0372**
30 min post-TSST (10)	DGA	Baseline	0.001142	0.001487	0.4434
	DGA	9	−0.0039	0.001487	0.0096 **
60 min post-TSST (11)	DGA	Baseline	0.001814	0.001507	0.2306
	DGA	9	−0.00312	0.001517	**0.041**
90 min post-TSST (12)	DGA	Baseline	0.001689	0.001493	0.2596
	DGA	9	−0.00298	0.00152	**0.0516**
120 min post-TSST (13)	DGA	Baseline	0.002341	0.001492	0.1186
	DGA	9	−0.00193	0.001488	0.1955
Pre-TSST (9)	TAD	Baseline	0.000565	0.001402	0.687
	TAD	9	−0.00013	0.001438	0.93
30 min post-TSST (10)	TAD	Baseline	0.000172	0.001402	0.9023
	TAD	9	0.000992	0.001438	0.4908
60 min post-TSST (11)	TAD	Baseline	−0.00139	0.001402	0.3228
	TAD	9	0.000077	0.001439	0.9571
90 min post-TSST (12)	TAD	Baseline	−0.00083	0.001405	0.5551
	TAD	9	−0.00017	0.001442	0.9073
120 min post-TSST (13)	TAD	Baseline	−0.00106	0.001406	0.4501
	TAD	9	−0.00041	0.001439	0.7742

* The significant diet group–week–saliva sample–Δ carbohydrate interaction suggested the nature of the association between cortisol and Δ carbohydrate differed among diet groups (DGA, *N* = 22; TAD, *N* = 21), intervention week, and saliva samples. Therefore, we also used the mixed model procedure, accounting for repeated measurements, to estimate coefficients (slopes) for the relationship between log salivary cortisol (nmol/L) and magnitude of change (Δ) in carbohydrate consumption from the pre-intervention (habitual) to the diet intervention period as a continuous variable at each combination of diet group, intervention week, and saliva sample. The *P*-value in bold indicates that the slope was significantly different from 0, while the double asterisk (**) indicates that the slopes for that week and sample number significantly differed between the DGA and TAD groups. Baseline age, education, BMI, and hunger score from the Three-Factor Eating Questionnaire were included as independent variables in the statistical model. TSST: Trier Social Stress Test; Pre-TSST: Saliva sample taken immediately prior to the stress test; DGA: Dietary Guidelines for Americans diet intervention group; TAD: typical American diet intervention group.
